# Wave-like patterns in parameter space interpreted as evidence for macroscopic effects resulting from quantum or quantum-like processes in the brain

**DOI:** 10.1038/s41598-022-22661-8

**Published:** 2022-11-07

**Authors:** Stoyan Kurtev

**Affiliations:** Alliance Manchester Business School, The University of Manchester, Booth Street West, Manchester, M15 6PB UK

**Keywords:** Human behaviour, Quantum mechanics

## Abstract

Data from eight numerosity estimation experiments reliably exhibit wave-like patterns in plots of the standard deviations of the response times along the abstract parameter of the magnitude of the error in the numerosity estimation. An explanation for this phenomenon is proposed in terms of an analogy between response times and error magnitude on one hand, and energy and position of quantum particles on the other, constructed using an argument for an overlap between the mathematical apparatus describing Hopfield-type neural networks and quantum systems, established by some researchers. Alternative explanations are presented within the traditional explanatory framework of oscillations due to neural firing, involving hypothetical mechanisms for converting oscillation patterns in time to oscillation patterns in the space of an abstract parameter, such as the magnitude of the error during numerosity estimation. The viability of the proposal of causal influences propagating from the microscale of quantum phenomena to the macroscale of human behavior, needed for the first type of explanation, is exemplified by the phenomenon of magnetoreception in some species of birds, which is allegedly quantum in nature.

## Introduction

The question of whether quantum phenomena at the microscale in the brain play any role in influencing or even determining behavior at the human macroscale of experience is a controversial one^1,2^. Some researchers have proposed that quantum models of decision making fit experimental data better than classical models^1,3^, without suggesting physical causality from the microscale to the to the macroscale as possible explanation for this finding^1,4^. This avenue of research is labelled “quantum cognition”, and it is interested in applying principles and methods from quantum physics to the study of cognition as an abstract system, without concerning itself with the viability of the physical instantiation of the proposed quantum models in the brain. There are also several other claims about the possible existence of quantum phenomena in the brain that allegedly serve as the physical correlate of consciousness^5–7^, collectively referred to as the “quantum brain” hypothesis, but none of them has earned widespread acclaim.

The standard objection to the first class of theories—those that conceptualize quantum-like phenomena in observed behavior as abstract models of cognitive function—is that they model phenomena with a more complex conceptual and mathematical apparatus, which could accidentally cover a broader spectrum of cases (see^1^, section 4.3). For that reason, it is not clear what necessitates the use of intuitively unrealistic mathematical constructs to model human thought and behavior. The objection to the second class of theories is that, to the best of current knowledge, it is impossible to realize quantum phenomena in the hot, wet brain at macroscopic spatial and temporal scales far beyond the scales at which they are observable in physics experiments^8^. The widely held assumption among researchers, therefore, is that quantum phenomena are unlikely to play any role in human cognition and the phenomenon of consciousness.

This article describes a reliably reproducible effect in human behavioral data which is used as an argument for the possibility that quantum phenomena at the microscale in the brain can manifest themselves on the macroscale of human behavior. The argument is based on commonality in the mathematical modelling approaches to quantum systems and neural networks and the presumed existence of a causal mechanism allowing some bird species to sense the orientation of Earth’s magnetic field via quantum effects occurring on the microscale.

### The functional similarity between quantum systems and neural networks

Artificial neural networks (ANNs) are commonly regarded as idealized models of the biological neural networks in the human and animal brains, and recently it has been established that in some cases the mathematical formalisms describing specific types of neural networks and some basic aspects of the functioning of quantum systems are equivalent. For example^9^ and^10^ showed that the Schrodinger equation expressed as eigenwaves in Feynman’s path-integral formalism and the dynamics associated with it is mathematically equivalent to a Hopfield-like associative neural network that can realize cognitive functions like memory, pattern recognition and recall. Neural networks of this type are regarded as biologically plausible, as pointed out already in the original paper proposing this specific ANN^11^.

In this paper, John Hopfield introduces also the notion of “energy” of the global state of the network, by analogy with the energy of physical systems, and shows that the network can perform useful tasks, such as recall of a trained state, pattern recognition, generalization, etc., by settling into a local state of minimal energy via gradient descend along the energy parameter. More recent work^12^ suggests that in this type of ANNs, the “quantumness” implied by the overlap of the mathematical apparatus describing the quantum systems and the neural network, emerges naturally under certain conditions, and the “energy” term in the latter corresponds to the energy term in the former (see also^13^ as another example), i.e., the analogy spotted by Hopfield between the parameter he defined for his neural network and physical systems seems to be a deep one, prompting the authors of^12^ to suggest that the physical reality itself could be a neural network at the most fundamental level of description.

If we turn this suggestion around, we could think of at least some biologically plausible neural networks, and even the conscious mental state in the human brain, as a quantum system. Aside from the question of the physical realizability of sustained quantum entangled states in the physical environment of the human brain, the mathematical equivalence quoted above suggests that the human brain and the conscious mental state it sustains can be regarded at least as a quantum-like system at an abstract level (a view espoused in general within the “quantum cognition” field of research), if not as a physical quantum system as understood within physics. That view has consequences regarding the outcomes of cognitive psychology experiments and would predict different outcomes than traditional cognitive psychology theories, by analogy with the distinction between classical and quantum effects in physics.

The most defining characteristic of “quantumness” in physics phenomena is the discreteness of the values that certain parameters can obtain, which also gives the name to this type of physics theories. The discreteness is usually characterized by wave patterns in plots of experimental data and their corresponding theoretical models, arising from the Schrodinger wave equation, whose solution sometimes has discrete eigenvalues. Such wave patterns, on the other hand, are rarely observed in cognitive psychology data, and are invariably associated with variations in a measure of performance in time (see^14^ for a review). There are cases where oscillations in time are modelled with a quantum mathematical formalism, such as^15^, but in those cases the authors do not make the claim that the cognitive processes they model are physically quantum in nature. The occurrence of a wave pattern would be unexpected in a measure of response times or error rates along a perceptual parameter, rather than in time. Such parameters could be length, size, auditory pitch, color, etc., i.e., perceptual states where the stimulus is experienced as the same type of percept along a gradient of different magnitudes of the same sensory stimulation. A wave pattern there would be unexpected within traditional cognitive models, because performance is expected to change monotonically with changes in the perceptual parameter, while it would arise naturally under the proposed assumption of quantumness of the cognitive mental state.

One should note that in order to establish the existence of a wave pattern, the perceptual parameter needs to be sampled at sufficiently many values of the parameter—at least 4, and preferably well over 4, which would allow multiple cycles of the wave pattern to be revealed.

### Experimental paradigm for probing the “quantumness” of mental states

Behavioral experiments typically measure accuracy and response times. Fortuitously, those two measures of performance can serve as analogues of the measures of position and momentum relating to physical particles. Accuracy, in paradigms involving numerosity or its analogues, can be construed as a monotonous measure of distance in mental space, where distance is represented by the difference between the correct answer and the actual response, i.e., the magnitude of the error. Response time, in turn, indicates how long it takes for the mental state to transition from the initial perceptual state of observing the stimulus to the subsequent action state of pressing the button to give the response. In that way, it is a proxy measure of how long it takes to make the decision within the particular experimental paradigm, which is typically selection of the response among two or more alternatives. One can think of this process of response selection as a transition through mental space, starting at the location representing the initial perceptual state and ending at one of several alternative locations, representing the alternative choices. The time it takes to make this transition is indicative of the “energy” of the mental state expressed as a difference in some abstract measure of brain activity between the initial perceptual state and the final decision state, analogously to the gradient descend from a higher energy state to a local minimum in the Hopfield network. Because of the mathematical equivalence of the energy parameter in the Hopfield ANN framework and the energy parameter of quantum systems, the response times would reflect also quantum-like perceptual information processing at the level of neural firing, if we assume no causal effects from the microscale of individual particles to the macroscopic scale of neural firing. If we go further and assume that the microscale of quantum phenomena plays a role in conscious mental activity, the response times would reflect information processing at the microscopic quantum scale with causal chains propagating down from the perceptual mechanisms of the sense organs to the microscale constituting the perceptual experience, quantum information processing at the microscale constituting the decision making and conscious thought, and causal chains propagating up the spatiotemporal scale for actuating the motor response. In summary, the magnitude of the error and the response time in a typical cognitive task could be proxy measures for conscious states physically realized in the brain either by quantum-like macroscopic neural network parameters analogous to position and momentum, or by the actual positions and energies/momenta of individual particles of brain matter on the microscale.

A typical numerosity estimation experimental paradigm, where participants are asked to estimate the number of objects of the same type, e.g., dots, shown on the screen, is in that way analogous to probing measures of position and momentum along a single spatial dimension formed by an abstract parameter. The probability density of finding the mental state in a specific location in that dimension, formed by the magnitude of the difference between the actual number of objects in the stimulus percept and the estimate, is given by the count of responses falling in that location, in the case of a free choice answer. A proxy measure of the probability density is the standard deviation of the responses within a range of values, in case of a continuous variable such as a very finely measured distance between the perceptual state and the response, and also the standard deviation of the response time. The standard deviation is the most appropriate proxy measure for probability density of all statistical momenta of a distribution, due to the fact that it is inversely proportional to the density of grouping of the values within a fixed interval. The mean does not reflect the density of the grouping of the values in a distribution, and the higher order momenta reflect finer details in the properties of a grouping, rather than the general property of its density. Thus, the probability density of position is given by the counts of the responses for each value of the differential between the actual number and the estimate, while the probability density of momentum is given by the counts and also by the standard deviations in the histogram of the response times, suitably segmented into periods with the same length.

Free choice paradigms produce unequal distribution of the counts of the responses because the correct answer is most likely to be selected and the probability of selection decreases monotonically with increasing distance from the correct answer. Equal distribution of the counts can be enforced by asking the participants to estimate the numerosity of the object compared to a predefined number by offering them only two choices—higher or lower than that number. In that case, the counts for each value of the difference between the actual number of dots and the target number can be counterbalanced, at the expense of being unable to estimate the probability density of position using the counts. However, that paradigm makes it possible to measure the probability density distribution of the “energy” of the mental state by plotting the standard deviation of the response times associated with each value of the difference along the abstract dimension.

Eight experiments employing this type of experimental paradigm are presented in this article. Four of them (Exp I, Exp II, Exp III and Exp IV) were specifically designed for the purpose of testing the above hypothesis and performed by the author. The other four—OS Exp I^16^, OS Exp II^17^, OS Exp III^18^ and OS Exp IV^19^—were designed and performed for other purposes by other researchers, who made the data freely available under the Open Science Framework for data sharing and collaboration, but fit the requirements for an experimental paradigm allowing the observation of patterns in a behavioral measure along a single dimension, as outlined above.

All experiments involve a task of estimating the number of dots presented on the screen. Although the task was different in each of the experiments, the results are comparable for the purpose of testing the hypothesis presented above, because they all allow calculating the standard deviations of the response times for a range of consecutive differentials. The paradigms vary in whether the response is free entry of a number or comparison against a target number and the ranges of numerosity of the stimuli, resulting in different distributions of the numbers of trials and the response times associated with each numerosity (see Table 1, for more details on the methods see Appendix A).

The OSF experiments are an opportunistic sample obtained by searching by keywords in online data repositories, such as OSF.io, figshare, Mendeley, etc. In total 22 datasets were identified and processed using the same method, and 4 of them were found to have an experimental paradigm sufficiently similar to those used by the author. No dataset was excluded from consideration because of absence of the reported wave-like pattern effect.

**Table 1:** Main differences in the methods of the eight experiments.

Experiment	Response type	Numerosity range
1. Exp I	Free entry (three-choice)	40–69
2. Exp II	Two-choice	40–69
3. Exp III	Two-choice	40–69
4. Exp IV	Two-choice	40–69
5. OS Exp I	Free entry (knob)	10–99
6. OS Exp II	Two-choice	30–170
7. OS Exp III	Free entry (keyboard)	10–20
8. OS Exp IV	Two-choice	10–44

Counts distribution	Average response time (ms)	STD distribution
1. Bipartite exponential	874	Flat
2. Flat	890	Peaked
3. Flat	877	Peaked
4. Flat	1745	Peaked
5. Bell shaped	2312	Flat
6. Bell shaped	998	Flat
7. Bell shaped	5611	Flat
8. Flat	2042	Peaked

### Ethics declaration

Ethics approval was obtained for all four experiments performed by the author from the ethics committee at Coventry University, United Kingdom. The reference numbers of the approvals are P36680 for experiment 1, P48127 for experiments 2 and 3, and P88672 for experiment 4. The research was conducted according to the principles expressed in the Declaration of Helsinki. Informed consent was obtained from each participant at the beginning of the experimental session.

## Results

**Figure 1:**
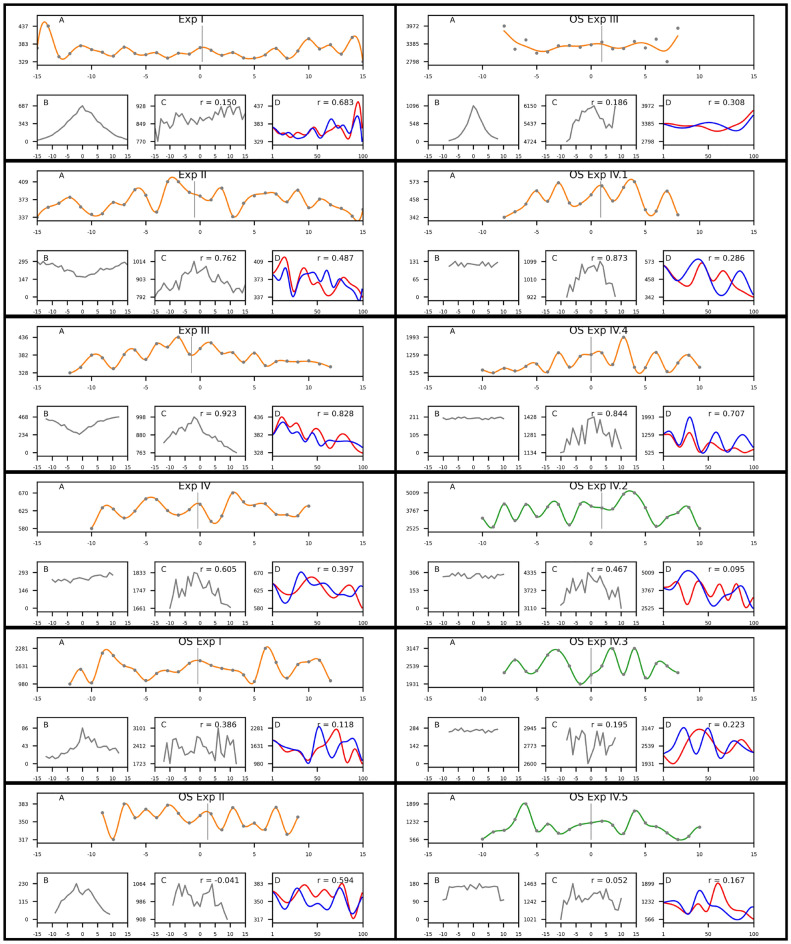
(Top to bottom, then left to right) Panels for the Standard Deviations (**A**), Counts (**B**), Averages (**C**) and superimposed Standard Deviations (**D**) of the response times (in milliseconds, vertical axes) plotted by the difference between the actual number of dots and the estimate (horizontal axes) in the eight experiments. Panel (**D**) shows the interpolated standard deviations for the two arms (positive [blue] and negative [red] differences, interpolated with resolution 100 points) and the correlation coefficient for the two arms of the plot. The grey line near the middle in panels A indicates the axis of symmetry used to separate the two arms. The r values in panels D in the first 9 plots (orange) are mostly moderate to high due to the symmetry of the plot, with the exception of OS Exp I where the wave-like patterns are somewhat misaligned, while the r values in the last 3 plots (green) are low due to asymmetry of the patterns.

In all eight experiments the pattern of the standard deviations of the response times when plotted by the difference between the actual numerosity of the dots in the visual stimulus and the estimated numerosity (or the target number in some of the paradigms) exhibits an ostensible wave-like shape (see panel A in Fig. 1, which shows the values of the standard deviations as grey dots and an interpolation of the intermediate values with a univariate smoothing spline of degree 3, as defined in the SciPy package for Python). What is more, there is a close match between the two arms of the plot—the pattern for negative differences and the pattern for positive differences (panel D in Fig. 1). The closeness of the match was evaluated numerically by correlating the interpolated plots for the two arms using Pearson product-moment correlation, as defined in the NumPy package for Python, on a segmentation of each arm into 100 sample points.

As shown in panels D in Fig. 1, the correlations between the patterns in the two arms of the plots are moderate to high for the first 9 datasets marked in orange, ranging from r = 0.285 to r = 0.828, mean = 0.490 (except in OS Exp I, which has r = 0.118 due to misalignment of the peaks), and indicating a close match between the two arms of the plots. The last 3 datasets marked in green are from atypical populations and the match between the two arms of the plot is weaker (r ranging between 0.095 and 0.223, mean = 0.160). Note that the measurement of RT from each trial goes into one of the data points in the plot, meaning that the aggregate value in each data point is a result of events occurring independently from each other and from the events in the other data points. (It should be noted that there are some differences in the data processing methods used for the eight datasets—see Table 2).

**Table 2:** Main differences in the data analysis for the eight experiments.

Experiment	Number of valid trials	Trials per condition	Mean # of trials per condition
1. Exp I	8604	30–690	278
2. Exp II	7248	165–295	234
3. Exp III	9312	240–470	372
4. Exp IV	5167	210–290	246
5. OS Exp I	934	12–86	37
6. OS Exp II	2450	30–230	129
7. OS Exp III	6630	25–1100	390
8. OS Exp IV	2040–5836	100–300	120–278

Outliers ignored at	Error responses ignored	Percent trials ignored (%)	
1. 1.96 std (global)	N/A	4.8	
2. 1.96 std (within participant)	Yes	28.5	
3. 1.96 std (within participant)	Yes	30	
4. 1.96 std (global)	No	5.5	
5. 3 std (global)	N/A	2.5	
6. 1.96 std (global)	No	4.8	
7. 1.96 std (global)	N/A	4.7	
8. None	No	None	

The response time measures have been calculated with outliers > 1.96 standard deviations from the global or the participant mean removed, except in two experiments. This eliminates only a small number of trials and makes the shapes cleaner. In two of the experiments incorrect responses are also removed from the calculations, resulting in the removal of substantially larger proportions of all trials.

In Exp I, OS Exp I, OS Exp II and OS Exp III the counts are unbalanced, with more responses in the difficult conditions and fewer responses in the easier conditions, resulting in a peaked (bipartite exponential) curve. In the other experiments, the responses are closely balanced due to the counterbalancing of the conditions, with somewhat fewer responses in the difficult conditions in the cases of selection of only the correct responses.

In Exp I, OS Exp I and OS Exp III the responses are open-ended, i.e., participants are entering a number, while in the other experiments they are comparing the numerosity of the dots against a target number and making a binary choice. In OS Exp I, OS Exp III and OS Exp IV the response times are very slow (over 2000ms).

OS Exp IV involves five different types of populations. Datasets 1 and 4 are from typical populations (Western adults and children respectively) as all other experiments reported here, while datasets 2,3 and 5 are from atypical populations (indigenous people, preschoolers and dyscalculics respectively).

The r values in panel C are calculated from the actual values in the two arms of the plot and are displayed for comparison. The mean r value for the means is 0.521, ranging from − 0.041 to 0.923, suggesting that they are unreliable. The high r values in all experiments except the last 3 are mainly due to the peaked shape of the distribution.

In addition to the correlation analysis, computational modelling was performed to assess the probability of obtaining the observed correlations from random distributions of the data points (the values for the standard deviations in panel A). To that end, the data for the 8 plots in panels A of Fig. 1 were normalized by linear scaling within the interval [0,1] and random distributions for the data points of each plot were generated drawn from a normal distribution with mean = 0.44, SD = 0.27, which are the parameters of the aggregate set of all data points from the 8 plots. Each random distribution was interpolated in the same way as for the actual plots in panels D, and the correlation between the two arms was computed with the midpoint separating the two arm set at 0. This procedure was repeated 100,000 times and the proportion of correlations higher than the actual correlation displayed in panel D was calculated as a percentage of all 100,000 obtained values. The resulting values can be thought of as the probability of obtaining the hypothesized effect of closely matched wave-like patterns, quantified via the correlation values, by chance. For the first 9 datasets, where the effect is expected to be present, the probabilities calculated with this procedure were: 0.00402, 0.0514, 0.00077, 0.17541, 0.4144, 0.06861, 0.27772, 0.30994, 0.01718. For the last 3 datasets, where the effect is expected to be absent, the probabilities were: 0.45863, 0.36414, 0.38227. Finally, a two-sample equal variance (homoscedastic) two-tailed *T*-test with alpha level of 0.05 was performed on the obtained probability values, showing that the two types of simulated datasets differ significantly in the probability of obtaining the wave-like pattern by chance (t(10) = − 2.740, p = 0.021). This result reinforces the conclusion from the correlation analysis that the regularity of the patterns in the first 9 datasets is unlikely to be accidental. (The code for the computational modelling procedure is available in the “Data availability” section).

## Discussion

The results from the eight experiments presented in this article suggest the existence of regular wave-like patterns in some measures of performance when plotted along an abstract dimension. This artificial dimension is constructed from multiple points that sample a parameter attributed to different instantiations of the same mental state, in this case different perceptual states of numerosity or, more generally, magnitude.

The existence of wave-like patterns in measures of performance and measures of brain activity is unsurprising and is well established. There are perceptual cycles unfolding in time, established with different paradigms (see^14^, also^20^ and^21^), typically ascribed to cyclical brain activity—neural oscillations, i.e., temporal cycles of variable neural firing rates. The surprising finding in the presented set of experiments is that the wave-like patterns occur along an abstract parameter dimension, rather than a temporal dimension, where they can readily be explained by neural oscillations. While it is possible to construct an explanation that proposes a mechanism for translating temporal neural oscillations into oscillations in abstract space, the physical realizability of such a mechanism in the noisy system of neural firing in the human brain remains questionable.

A possible mechanism for converting temporal oscillations into variations in an abstract parameter of the mental state is offered by the phenomenon of travelling waves discovered in the rat neocortex (see, e.g.^22^), the cat neocortex^23^ and even in human cortex^24^. To that end, it can be hypothesized that the temporal oscillations pass through premotor cortex and affect the execution of the finger movement during the response. More specifically, the phase of the oscillatory activity would disrupt or focus the neural firing triggering the execution of the finger movement, leading to less consistent or more consistent timings of the movement. The second assumption that needs to be made in this construct is that conceptual states representing different magnitude are somehow topologically mapped in the cortex. More specifically, the neural representations of gradually increasing magnitude need to form a gradient along some cortical area or in the state space of a parameter governing the triggering of neural firing. That mechanism needs to perform reliably in order to convert temporal oscillatory patterns into variations in an abstract parameter governing finger movements.

Another mechanism that could explain these wave-like patterns can be based on the phenomenon of oscillations due to the activity of grid cells (see^25^). The grid cells have oscillatory patterns that depend on an abstract parameter, such as regularly spaced locations in physical space, and are a good example of how temporal oscillatory phase can be converted to abstract parameter phase and vice versa. The oscillatory phenomenon has been detected also in abstract space in a paradigm of comparing the lengths of two line segments, similar to those presented in this article (see^26^). A computational model study has demonstrated that grid cells arise naturally in this type of tasks^27^.

These explanations concern brain mechanisms and activity on the macroscopic scale. They can explain the finding of wave-like patterns in abstract mental space presented in this article with some additional assumptions. The advantage of this approach is that it does not assume any additional cognitive mechanisms beyond the standard ones used in the literature on cognitive processes in the brain. However, if we admit the possibility of either quantum-like or physically quantum information processing in the brain, then the conversion of temporal oscillations into oscillations in an abstract parameter is resolved naturally through the relation between the wave patterns in the probability density of position and momentum, known from quantum physics. The main outstanding issue within this framework is the possibility of causal influences going across 10 orders of magnitude on the spatial scale from the domain of individual particles with sizes on the order of picometres ($10^{-12}$ m) to the size of brain structures on the order of centimeters ($10^{-2}$ m). To resolve it, we need to assume that the measurements of macroscopic events, such as finger movements to press a button, are causally connected to phenomena at the microscopic scale, such as the probability of finding a particle (an atom, or maybe even an atomic nucleus or an electron) in a particular state. If such mechanism exists, then it would be conceivable that the pattern of probability density in the particle’s configuration space (or that of an entangled ensemble of particles), manifests itself in a similar, related pattern in the measurements of macroscopic behavior. More specifically, the wave-like pattern of variability of the response times in the abstract space of a single-parameter magnitude of the perceived stimuli, which is analogous to probability density, could be a manifestation of the wave-like distribution of probability density of position or momentum, or any other parameter in the configuration space of the state of a particle, at the microscopic scale. That hypothesis, in fact, does not preclude the existence of the classical mechanisms presented above; they could still be present in the brain, but in this case they would merely be intermediary links in the causal chain of physical interactions from the microscale to the macroscale.

The feasibility of a mechanism relating properties on the microscale to behavior on the macroscale has traditionally been questioned by physicists, given the vast difference in orders of magnitude between those two scales—approx. $10^{10}$ or $10^{9}$. However, there is an example of such a mechanism that has been discovered recently, namely, magnetic field sensing in some birds’ brains, where a quantum effect on the microscale affects the behavior of birds and allows them to navigate in space (see^28^). That yields credibility to the idea that similar “sensing” could be occurring in the human brain when trying to evaluate a complex perceptual stimulus in a challenging task. In this case, the sensed property would not be the orientation of the magnetic field (which is in fact converted to relative probability of two particular chemical reactions taking place in the bird’s brain), but the landscape of parameter space related to the microscopic physical correlates of magnitude and/or effort on the microscale in the human brain.

## Data Availability

The datasets generated and/or analysed during the current study are available in the OSF.io repository, https://osf.io/n5feg/, as well as the Python code for producing the plots and the computational modelling with random distributions of the data points.
